# An unusual dermoid cyst in subcutaneous tissue of the mastoid region: A case report

**DOI:** 10.3892/etm.2013.1080

**Published:** 2013-04-26

**Authors:** DAQING ZHAO, YU HAN, YANG CHEN, JIANHUA QIU

**Affiliations:** Department of Otolaryngology - Head and Neck Surgery, Xijing Hospital, Fourth Military Medical University, Xi’an, Shaanxi 710032, P.R. China

**Keywords:** dermoid cyst, mastoid, subcutaneous tissue, lymph node

## Abstract

Dermoid cysts of the head and neck are rare lesions. An unusual dermoid cyst in the subcutaneous tissue of the mastoid region is easily confused with lymph node abnormalities; therefore, diagnosis and treatment are often challenging. We describe a 22-year-old male with an unusual dermoid cyst in the subcutaneous tissue of the mastoid region whose only complaint was a retroauricular lump, which was similar to an intumescent retroauricular lymph node. Final diagnosis was confirmed by pathological examination of the surgically removed specimen. A dermoid cyst in the subcutaneous tissue of the mastoid region is rare and may be misdiagnosed as a retroauricular lymph node. A lump may be the only presenting symptom without hearing loss and vertigo complaints. Complete excision of the tumor must be ensured and pathological analysis is important for the diagnosis.

## Introduction

A dermoid cyst is a rare benign tumor, which is principally situated close to the midline of the body. It is a developmental lesion, histologically composed of ectoderm and mesoderm; however, it has no endoderm. In approximately 7% of cases, these cysts affect the head and neck region; they are frequently encountered in the area of the lateral eyebrow, the orbit and the nose (>80%), with the remainder located in the neck, occipital or frontal midline, lip or palate (1). However, little is known about dermoid cysts in the subcutaneous tissue of the mastoid region. We report a case of a dermoid cyst in the subcutaneous tissue of the mastoid region without hearing loss and vertigo, which was misdiagnosed as an intumescent retroauricular lymph node. The location of this tumor and its clinical features make this a unique case.

## Case report

The patient was a 22-year-old male presenting a lump that had grown gradually under the skin of the right mastoid for 2 years. The patient denied any hearing loss or vertigo. There was no pain and no inflammation in the lump. The patient was healthy, with the exception of the lump. The patient had been assessed at the community hospital one year earlier and was treated with antibiotics, which were ineffective. The patient then came to Xijing Hospital (Xi’an, China) for further evaluation and treatment.

On examination, the two ears appeared normal. The patient had normal and symmetrical facial and cervical structures with no deformities of the pinnae or external auditory canals. The ear drums were normal and no air-fluid levels or bubbles were observed behind the drum. Impedance audiometry and audiogram were also normal. There were no significant findings in the remainder of the head and neck examination, with the exception of the tumor, which was initially considered a retroauricular lymph node.

**Figure 1.**
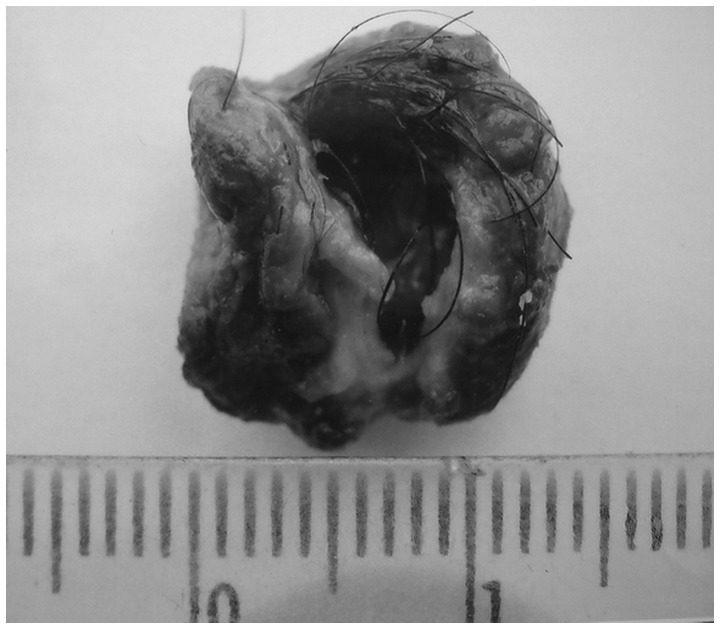
Surface of the tumor was granulated and a number of hairs were observed in the tumor.

Surgical biopsy was performed with the patient under local anesthesia. When the skin flap was opened, a pink encapsulated mass was observed occupying the right mastoid surface. The mass extended from the subcutaneous tissue to the mastoid cortical plate without destruction of the mastoid bony wall. The tumor and mastoid cortical plate were umbilicated in the mastoid cavity. The mastoid cavity maintained integrity. The surface of the mass was granulated and a number of hairs were observed in the tumor when it was slivered. The diameter of the tumor was approximately 1.3×10^−2^ m (Fig. 1). Following complete removal of the tumor, the defect was reinforced with muscle and fascia.

**Figure 2.**
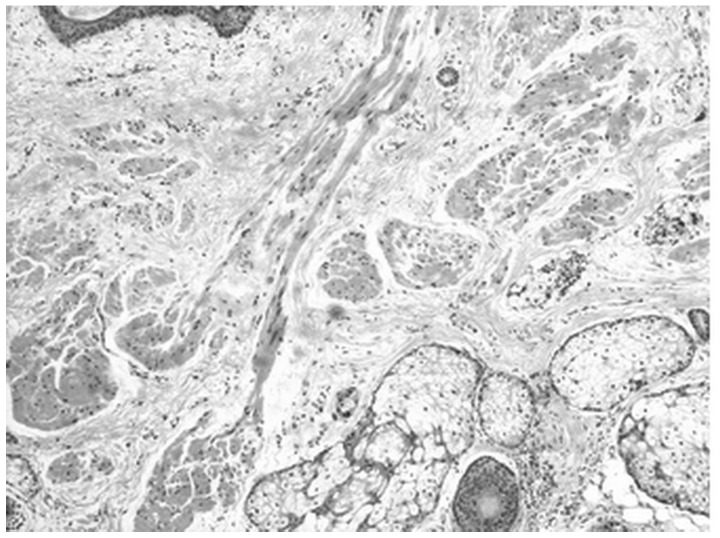
Histopathological appearance of the tumor (H&E stain; magnification, ×100). The interstitial tissue contains squamous epithelium, muscle, hair roots, sebaceous glands and sweat glands. H&E, hematoxylin and eosin.

Pathological analysis confirmed the diagnosis that the tumor was a dermoid cyst (Fig. 2). Two years after surgery there was no evidence of any recurrent tumor in the region of the right mastoid. All studies were performed under the consent of the patient and with approval from the Human Studies Committee of the Xijing Hospital of the Fourth Military Medical University.

## Discussion

Dermoid cysts are tumors composed of two germ layers, ectoderm and mesoderm. They are epithelial-lined cavities with skin appendages, including hair, hair follicles and sebaceous glands. This distinguishes them from cholesteatomas, which are composed only of ectodermal elements, and teratomas, which are composed of ectodermal, mesodermal and endodermal elements.

Dermoid cysts may occur anywhere in the body. They primarily occur in the gonads; however, they also occur at extragonadal sites along the midline of the body. The head and neck region is a rare location for such tumors in children and adults. Therefore, the pathological evaluation and clinical management of these tumors is extremely difficult. Toynbee reported the first case of a dermoid cyst of the mastoid in 1866, when hairs were identified in the mastoid cavity surrounded by epidermis (2). Howie discovered a dermoid cyst in the middle ear of a 29-year-old female who presented symptoms of hearing loss and vertigo (3). Steel reviewed reports on dermoid cysts in the mastoid from 1866 to 1976 and identified four cases in the literature that referred to non-hair-bearing cysts in the mastoid. Steel also reported a 67-year-old male who had been treated for intermittently-active chronic otitis media 4 years, which was the result of a dermoid cyst in the middle ear (4). Fried and Vernick published a report on a patient aged 22 months who had a dermoid cyst of the middle ear and mastoid (5). Minatogawa *et al* reported on a 6-year-old female patient with a dermoid cyst in the middle ear and low-tone unilateral conductive hearing loss (6). Farris *et al* reported on an 8-month-old female who was the youngest patient with a congenital dermoid cyst of the middle ear with a moderate conductive hearing loss of the ear in 1998. It was suggested that congenital inclusion may be the cause of the majority of dermoid cysts in the head and neck (7). Scolozzi *et al* reported a case of a 1-year-old female who was initially seen with a cutaneous fistula of the frontotemporal region, which revealed an intracranial dermoid cyst (8). Due to the small number of patients reported, little generalization has been made about the presentation of dermoids of the mastoid and middle ear. No gender preponderance has been noted.

The patient in our study was unique in several respects and there are no previous reports of similar cases. The patient had no symptoms, with the exception of the histologically-confirmed dermoid cyst in the subcutaneous tissue of the mastoid region. Patients previously reported often had a dermoid cyst in the mastoid and middle ear and usually suffered from hearing loss and vertigo. However, in this case, the dermoid cyst extended from the subcutaneous tissue to the right mastoid, the mastoid cavity was undamaged and hearing loss and vertigo did not appear. Therefore, prior to pathological analysis, it was easily confused with an intumescent lymph node.

As with the majority of cysts, local recurrence at the primary site is common unless the entire wall of the cyst is removed. Therefore, complete excision of the cyst during surgery must be ensured.

Although uncommon, dermoid cysts must be considered in the differential diagnosis of lumps in the mastoid region. However, their pre-operative differentiation from a retroauricular lymph node is not easy. Complete resection of the tumor is required and pathological analysis is important for the diagnosis.
